# NIR Rapid Assessments of *Blumea balsamifera* (*Ai-na-xiang*) in China

**DOI:** 10.3390/molecules22101730

**Published:** 2017-10-16

**Authors:** Fu-Lai Yu, Na Zhao, Zhi-Sheng Wu, Mei Huang, Dan Wang, Ying-Bo Zhang, Xuan Hu, Xiao-Lu Chen, Lu-Qi Huang, Yu-Xin Pang

**Affiliations:** 1 Tropical Crops Genetic Resources Institute, Chinese Academy of Tropical Agricultural Sciences, Danzhou 571737, China; flyu@catas.cn (F.-L.Y.); huangmei122@126.com (M.H.); wang_dan1414@163.com (D.W.); zhangyingbo1984@163.com (Y.-B.Z.); mchuxuan@163.com (X.H.); hillowchan@hotmail.com (X.-L.C.); 2 School of Pharmacy, Shihezi University, Shihezi 832002, China; zn26140@163.com; 3 Key Laboratory of Xinjiang Plant Resources and Utilization, Ministry of Education, Shihezi 832002, China; 4 School of Chinese Material Medica, Beijing University of Chinese Medicine, Beijing 100029, China; 5 National Resource Center for Chinese Materia Medica, China Academy of Chinese Medical Sciences, Beijing 100700, China; huangluqi01@126.com; 6 Center for Post-Doctoral Research, China Academy of Chinese Medical Sciences, Beijing 100700, China

**Keywords:** *Blumea balsamifera*, NIR, *l*-borneol, total flavone, rapid assessments, green chemistry

## Abstract

*Blumea balsamifera* (*Ai-na-xiang*) is used as an important plant source of natural borneol, which is widely used in the pharmaceutical industry. The aim of this study was to establish the methods based on near infrared (NIR) spectroscopy for determining the geographical origin of *B. balsamifera*, as well as developing a method for the quantitative rapid analysis of the active pharmaceutical ingredients (APIs). A total of 109 samples were collected from China in 2013 and arbitrarily divided into calibration and prediction sets using the Kennard–Stone algorithm. The *l*-borneol and total flavone contents of the samples were measured by gas chromatography and ultraviolet-visible spectroscopy, respectively. The NIR spectra were acquired using an integrating sphere and a partial least squares (PLS) model was built using the optimum wavelength regions, which were selected using a synergy interval partial least-squares (SiPLS) algorithm. The root mean square errors of prediction of the *l*-borneol and total flavone models were 0.0779 and 2.2694 mg/g, with R^2^ of 0.9069 and 0.8013, respectively. A discriminant model to determine the geographical origin of *B. balsamifera* (Guizhou and Hainan) was also established using a partial least squares discriminant analysis method with an optimum pretreatment method. The prediction accuracy rate of the model was 100%. NIR spectroscopy can be used as a reliable and environmentally friendly method to determine the API and the origin of different *B. balsamifera* samples.

## 1. Introduction

Near-infrared (NIR) spectroscopy (12,500–4000 cm^−1^) has been used in combination with chemometric methods in the pharmaceutical industry to rapidly identify specific active pharmaceutical ingredients (APIs) [1,2,3,4] with minimal sample preparation, fast analysis time and environmentally friendliness. For example, Feng and Hu [1] used NIR reflectance spectroscopy to develop a new method for determining the API contents of roxithromycin and erythromycin ethyl succinate tablets from different manufacturers in China. The quantitative partial least squares (PLS) models described in these studies were highly robust and exhibited good specificity, linearity, accuracy and precision properties, demonstrating the feasibility of these models for the rapid analysis of pharmaceutical products from different manufacturers. Ito et al. [2] used NIR transmittance spectroscopy to analyze acetaminophen and caffeine anhydrate in intact bilayer tablets with an appropriate fluctuation range for the thickness of the tablets. Furthermore, the calibration models produced in this particular study exhibited good linearity and accuracy characteristics with a fluctuation range of 4.30 ± 0.06 mm. Farrell et al. [3] employed NIR to detect the API contents of different types of escitalopram tablets using model updating. Notably, the predicted results of the primary models correlated well with the results of a full calibration model formed under secondary conditions after they had been updated using a Tikhonov regularization. Wu et al. [4] used on-line NIR spectroscopy to monitor the extraction of *Lonicera japonica* and demonstrated that NIR technology may be used to detect trace amounts of chlorogenic acid in *Lonicera japonica*.

Gas chromatography (GC) has been used as a reference method to determine the volatile components of oils [5,6,7]. Volatile oils are the main active pharmaceutical ingredients in several Chinese medicines, including *Blumea balsamifera* (*Ai-na-xiang*), *Mentha haplocalyx* (*Bo-he*), and *Rhizoma Chuanxiong* (*Chuan-xiong*). Although GC is a highly sensitive analytical method, it generally requires additional steps for the processing of the samples, making it a time-consuming method. NIR spectroscopy has also been used for the qualitative analysis of different samples, including distinguishing their geographical origin [8,9,10,11]. Li et al. [8] reported that NIR spectroscopy may not only be used to quantitatively determine the active components of a specific material but that it may also be used to determine the geographical origin of Flos *Lonicerae japonicae* with prediction accuracies of 100% and 86.8% for two provinces in China. Bevilacqua et al. [9] used NIR spectroscopy coupled to chemometric classification methods as a tool for traceability of olive oil samples with designated origin. The result showed that after processing the spectroscopic data by partial least squares discriminant analysis (PLS-DA) evidenced a rather high classification accuracy than modeling (SIMCA) approach.

*B. balsamifera* (*Ai-na-xiang*) has been used in Chinese medicine for centuries to treat a variety of different ailments, including pain and diaphoresis, as well as being used to expel wind and remove dampness [12]. *B. balsamifera* is also used as an important medicinal plant source of natural borneol (*l*-borneol content above 85%). The process used to purify borneol flakes also results in the formation of Blumea oil, which is widely used in the pharmaceutical, cosmetic and fine chemical industries [13,14].

The APIs of *B. balsamifera* are volatile oils and flavonoids, such as *l*-borneol, quercetin and blumeatin [15], all of which have traditionally been determined by GC and ultraviolet-visible (UV-VIS) spectrophotometry [16]. However, there are currently no quality control methods available for the rapid analysis of *B. balsamifera*. A synergy interval partial least-squares (SiPLS) algorithm has been used to select specific wavelength regions to improve the robustness of different calibration models. For example, Wu et al. [17] reported the use of a SiPLS method to select variables for monitoring the amino acid concentration profile obtained during the hydrolysis of *Cornu bubali*. The results of this study demonstrated that SiPLS is effective for selecting specific variables and that SiPLS models can provide high levels of accuracy.

The aim of the study was to establish qualitative and quantitative methods based on NIR spectroscopy to identify the geographical origin of *B. balsamifera* samples and quantitatively determine the APIs of these materials. In this paper, we collected 109 samples from three of the main producing areas of China, including 31 samples from the Guizhou Province, 77 samples from the Hainan province and one sample from Guangxi Province. We then used a partial least squares (PLS) model together with wavelength regions selected by SiPLS to determine the critical quality parameters of *B. balsamifera*, *l*-borneol and the total flavones. The reference values of the critical quality parameters were measured by GC and UV-VIS spectrophotometry. The performance characteristics of this new model were assessed by chemometrics indicators to prove the reliable analysis of NIR technique *B. balsamifera* powder. PLS-DA was used to differentiate the different *B. balsamifera* samples based on their geographical origin. The prediction accuracy rate was used to assess the feasibility of the method.

## 2. Results and Discussion

### 2.1. Quantitative Analysis of the l-borneol and Total Flavone Contents of B. balsamifera by GC and UV-VIS Analyses

**Figure 1:**
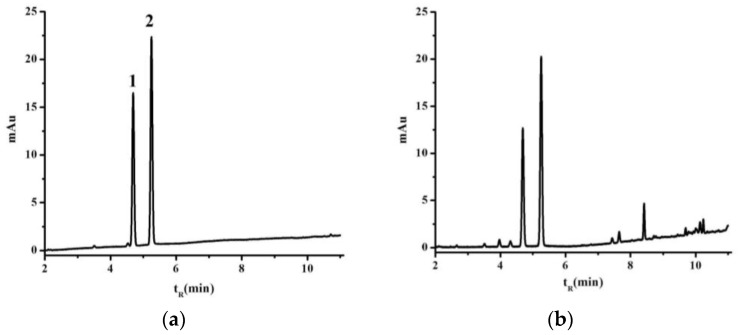
GC chromatograms of the *l*-borneol reference standard (**a**) and *B. balsamifera* solution (**b**). 1. *l*-borneol, 2. methyl salicylate.

**Figure 2:**
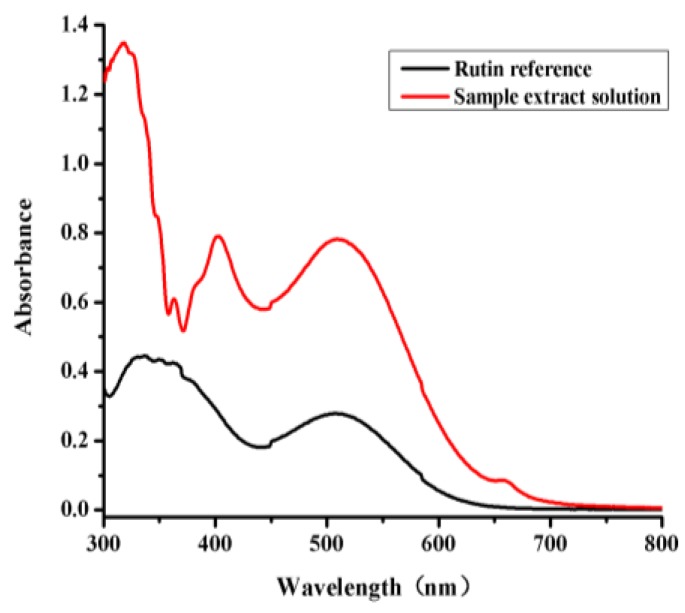
Full wavelength scans of a rutin reference and sample extract solution.

The GC and UV-VIS methods were fully validated before being used to test any of the samples. Typical GC chromatograms of an *l*-borneol and methyl salicylate (internal standard) reference standard and a sample of *B. balsamifera* solution are shown in Figure 1. The retention time of the *l*-borneol in the *B. balsamifera* solution was found to identical to that of the reference standard [16]. Rutin has also been reported as a suitable reference material for this type of analysis [18]. The UV-VIS traces of a rutin reference and a sample extract solution were investigated to measure the total flavone contents of the different *B. balsamifera* samples (Figure 2).

The key operating parameters and the calibration curves of the GC and UV-VIS methods were evaluated, and the results are listed in Table 1. Calibration curves were generated for *l*-borneol and the total flavones using concentration ranges of 10.371 to 207.428 μg/mL and 9.176 to 73.408 μg/mL, respectively, with 12 consecutive injections of six different concentrations. The calibrations curves exhibited good linearity characteristics within the specified concentration ranges. The content range of the measured samples extracting solution should be within the calibrations curves linear range. According to other validation parameters (i.e., repeatability, intermediate precision and recovery) these results also indicated that these GC and UV-VIS methods could be used as reference methods for the quantitative analysis of the *l*-borneol and total flavone contents in *B. balsamifera*.

Table 2 shows the statistical results for the analysis of the *l*-borneol and total flavone contents in *B. balsamifera*. The quantification results showed that the concentration ranges of *l*-borneol and the total flavones of *B. balsamifera* varied from 1.00 to 13.80 mg/g and 6.60 to 189.30 mg/g, respectively. The concentration range of the total flavones was therefore wider than that of *l*-borneol. Furthermore, the *l*-borneol content of *B. balsamifera* was much lower than that of the total flavones. However, *l*-borneol was determined to be the most abundant and active component of *B. balsamifera*, whereas the total flavones were the major non-volatile constituents. Thirty-one of the *B. balsamifera* samples were obtained from Guizhou Province, whereas 77 samples were obtained from Hainan Province. The average *l*-borneol and total flavone contents of the samples collected from Hainan were greater than those of the samples collected from Guizhou.

### 2.2. Special Features of the NIR Spectra and Outlier Selection

**Figure 3:**
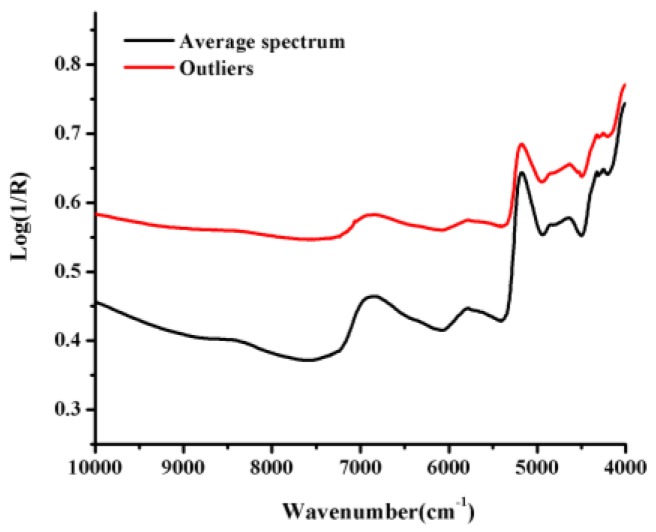
The average spectrum and the outlier NIR spectra of *B. balsamifera* samples from different origins.

The average spectrum and the outliers NIR spectra of *B. balsamifera* samples evaluated in the current study are shown in Figure 3. All of these spectra showed severe spectral overlap and baseline drift. In particular, we observed large fluctuations in the region of the first combination-overtone (FCOT, 7100–4900 cm^−1^) and combination region (CR, 4900–4000 cm^−1^). We also observed that the NIR spectrum of one of the samples was abnormal compared with most of other spectra, which indicated that it was outlying observation.

### 2.3. Spectral Pretreatment Processes and Determining the Optimum Latent Factor Numbers for the Calibration Models

We observed that the spectra were affected by the spectral noise, baseline drift and overlapping peaks (Figure 3). It was therefore necessary to eliminate all of the noise and interference factors using a series of appropriate spectral pretreatment methods to extract characteristic information pertaining to the pharmaceutical ingredients. We also investigated the effects of several preprocessing methods on the quantitative models, including standard normal variate (SNV), Savitzky–Golay smoothing (SG), multiplicative scatter correction (MSC) and Savitzky–Golay smoothing (SG) combined with derivative spectra. The optimum numbers for the latent factors were determined by the lowest predicted residual sum of squares (PRESS) value as well as PRESS plot, which was calculated using a leave-one-out cross-validation process. The first minimum value on a PRESS plot is usually used to determine the optimum number of factors with the best prediction. Besides, the number of latent factor cannot be too much to avoid over-fitting.

**Figure 4:**
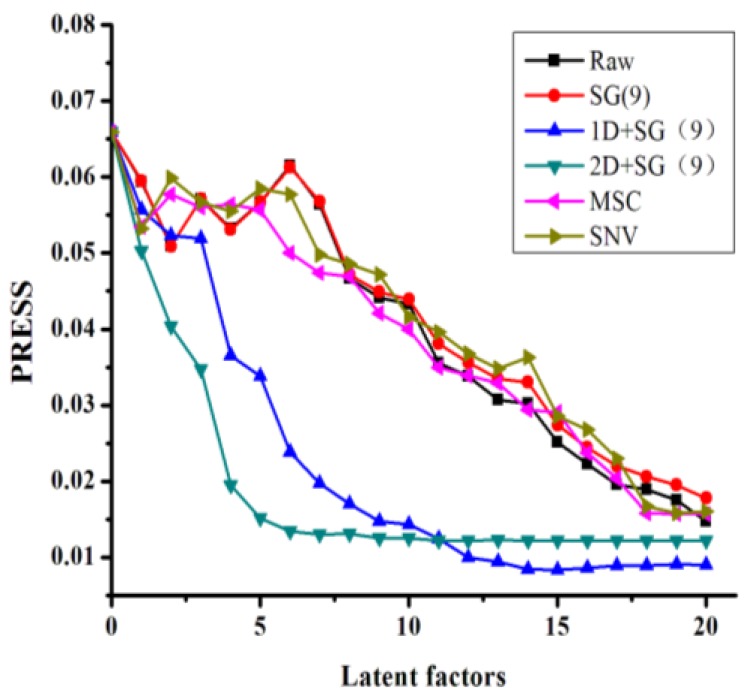
PRESS plot of *l*-borneol using different pretreatment processes. (Raw: raw spectra; MSC: multiplicative scatter correction; SNV: standard normal variate; SG: Savitzky–Golay filter; 1D: first derivative; 2D: second derivative).

Taking *l*-borneol as a representative example, Figure 4 shows the relationship between the latent factors and the PRESS value under different pretreatment conditions. These data show that increasing the latent factors leads to a reduction in the PRESS value. Compared with other pretreatment methods, there were several distinct advantages associated with the combination of derivative spectra with SG smoothing, which resulted in a sharp decrease in the PRESS value with eight latent factors. These results therefore demonstrated that the other pretreatment methods did not allow for the useful spectral information to be effectively separated from the overlapping spectra.

Table 3 shows the results of the PLS models for the total flavone and *l*-borneol contents of the different *B. balsamifera* samples, which were generated using a variety of different spectra pretreatment methods. The appropriate pretreatment methods were selected according to the results of a cross-validation process. The pretreatment of the derivative spectra in combination with SG smoothing showed that the PLS models of *l*-borneol had one of the lowest root mean square errors of calibration (RMSEC), root mean square errors of cross validation (RMSECV), of all of the pretreatment processes evaluated in the current study, with a coefficient of determination (R^2^) close to 1. This result was consistent with those shown in Figure 4.

### 2.4. Selection of the Wavelength Regions for the Calibration Models

The SiPLS algorithm was also applied to a calibration model to allow for the selection of a suitable wavelength region. The spectral set was split into different intervals and the optimum combination of sub-intervals was selected according to the lowest root mean square errors (RMSE). The parameters of the SiPLS algorithm had to be optimized to include the numbers of sub-intervals and combinations. The results of a previous report showed that the optimum parameters for the SiPLS algorithm included 20 sub-intervals and three sub-interval combinations. The SiPLS model used in the current study was therefore built using a random combination of three sub-intervals with 20 equidistant sub-intervals according to the results of this previous report [4].

**Figure 5:**
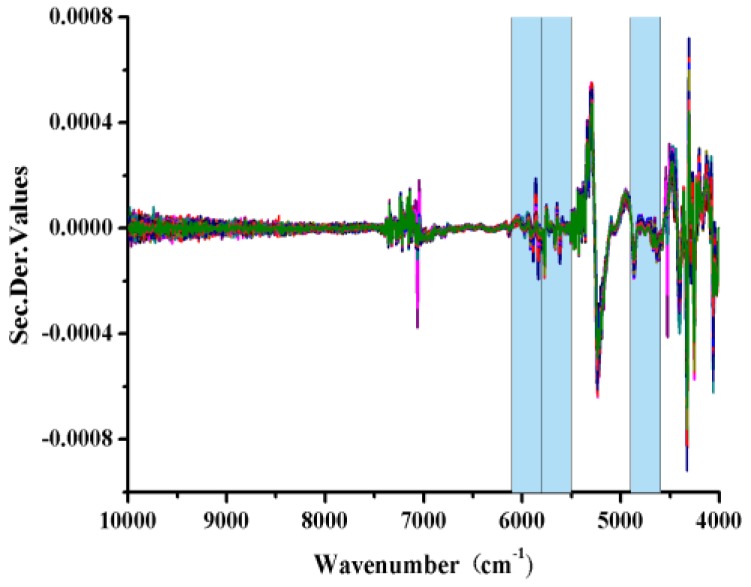
SiPLS-selected wavelength regions for the quantitative determination of *l*-borneol using a 2D + SG(9) pretreatment process.

The optimum SiPLS *l*-borneol model was built based on a combination of sub-interval numbers 3, 6 and 7 using seven factors, corresponding to 4601–4894, and 5504–6102 cm^−1^ (Figure 5). The optimum wavelength ranges for the calibration model of the total flavones were selected in the same way as 5805–6102, 7309–7606 and 8512–8809 cm^−1^.

### 2.5. Development and Validation of Calibration Models

The optimum model was determined based on the RMSEC, RMSECV, root mean square errors of prediction (RMSEP) and R^2^ values. Taking *l*-borneol as a representative example, the results in Table 4 showed that the SiPLS model of *l*-borneol with a second derivative (2D) + SG(9) pretreatment process provided one of the best performances.

**Figure 6:**
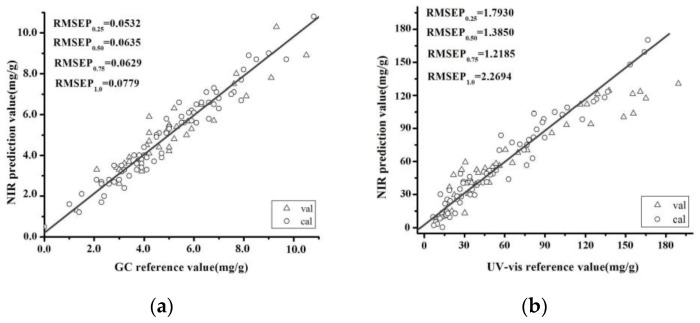
Correlation between the predicted and chemically determined values of *l*-borneol (**a**) and the total flavones (**b**) using a SiPLS model.

Furthermore, we propose to combine four RMSEP values to completely assess performance of model prediction. The quartiles are milestones in the population range (RMSEP_0.25_, RMSEP_0.5_, RMSEP_0.75_ and RMSEP_1.0_) that were calculated as:

(1) RMSEPnN=∑i=1n(yi−y𝑖^)2n

where $y_{i}$ and $\hat{y_{𝑖}}$ are measured and predicted response values for ith sample in validation. The N is the total sample number in validation and n ranges from 0 to N. We consider in particular n = 0.25 N, n = 0.5 N, n = 0.75 N and n = 1.0 N, and denote the corresponding RMSEP values by RMSEP_0.25_, RMSEP_0.5_, RMSEP_0.75_ and RMSEP_1.0_. The RMSEP_0.25_, RMSEP_0.50_, RMSEP_0.75_ and RMSEP_1.0_ values of the *l*-borneol validation set were 0.0532, 0.0635, 0.0629 and 0.0779 mg/g, respectively, which were very similar to those of the RMSEC. The R^2^ value of this validation set was 0.9069. A SiPLS model of the total flavones was also developed using the raw spectra. The RMSEP_0.25_, RMSEP_0.50_, RMSEP_0.75_ and RMSEP_1.0_ values of the total flavone validation set were 1.7930, 1.3850, 1.2185 and 2.2694 mg/g, respectively. The R^2^ value of this model was 0.8013. Figure 6 shows the results obtained for the *l*-borneol and total flavone contents using the SiPLS models. Notably, the values predicted by these models were close to those observed by GC and UV-VIS analysis.

### 2.6. Discriminant Analysis of B. balsamifera Samples According to Their Geographical Origin Using a PLS-DA Model

A discriminant model was established using a PLS-DA method to distinguish between the geographical origins of the different *B. balsamifera* samples. Table 5 shows the predicted results for the *B. balsamifera* samples using different pretreatment processes. The optimum number of latent factors and the predicted performances of the models were selected using different pretreatment methods. The use of a first derivative 1D + SG(9) and 2D + SG(9) pretreatment process gave a prediction accuracy rate of 100% based on an optimum number of latent factors of eight and four.

## 3. Materials and Methods

### 3.1. Plant Samples

The *B. balsamifera* samples used in this study (Table 6) were provided by the Tropical Crops Genetic Resources Institute, Chinese Academy of Tropical Agricultural Sciences (Danzhou, China). These materials were collected from different geographical regions of China, and were identified by Prof. Yu-xin Pang, according to their morphological characteristics and related documents [19,20]. Voucher specimens of these plants were also deposited at the Hainan Provincial Engineering Research Center for *Blumea balsamifera*, Danzhou, China.

### 3.2. Chemical Reagents

Standard samples of *l*-borneol and rutin were purchased from the National Institute for the Control of Pharmaceutical and Biological Products (Beijing, China). Methyl salicylate was supplied by the Tianjin Guangfu Fine Chemical Engineering Institute (Tianjin, China). Ethyl acetate, ethanol, sodium nitrite (NaNO_2_), aluminum nitrate (Al(NO_3_)_3_·9H_2_O) and sodium hydroxide (NaOH) were purchased from Xilong Chemical Corporation (Shantou, Guangdong, China). All of these reagents were purchased as the analytical grade. Deionized water was purified using a Milli-Q water purification system (Millipore Corp., Bedford, MA, USA).

### 3.3. NIR Measurement and Software

NIR spectra of the powdered plant materials were collected in the integrating sphere diffuse mode using an Antaris Nicolet FT-NIR system (Thermo Fisher Scientific Inc., Waltham, MA, USA). Each spectrum was collected over 64 scans in the range of 10,000 to 4000 cm^−1^ at ambient temperature with a resolution of 8 cm^−1^. Furthermore, each spectrum was recorded as Log(1/R) using air as a reference. Every sample was scanned once. All of the NIR spectra were collected and archived using the Thermo Scientific Result software. The Kennard–Stone (KS) algorithm was used to split the data sets into calibration and validation sets (2:1). The spectral preprocessing and model calculation steps were performed using the Unscrambler 9.7 software package (CAMO software AS, Oslo, Norway). The SiPLS algorithm toolbox was provided by Munck et al. [21].

### 3.4. Determination of the l-borneol Content by GC Analysis 

GC analysis was performed after the collection of the NIR spectra. According to the previous method [16], a small sample (2 g) of the material was extracted with ethyl acetate (25 mL) under ultrasonic irradiation (40 KHz, 400 W, KQ-500DE, Kunshan Ultrasonic Equipment Co., Kunshan, Jiangsu, China) at 30 °C for 30 min. The ethyl acetate extract was then passed through a Millipore filter (0.22 µm, Tianjin Jinteng Laboratory Equipment Co., Tianjin, China) prior to being analyzed by GCs.

The filtered extracts were analyzed on an Agilent 7890A gas chromatograph equipped with a flame ionization detector (FID) and an Agilent G4513A automatic sampler (Agilent Technologies, Santa Clara, CA, USA). A HP-5 quartz capillary column (30 m × 0.32 mm) coated with a 0.25 µm film (5% phenyl methyl siloxane, Agilent) was used to analyze the samples. The column temperature was maintained at 80 °C for 2 min after injection, and then programmed to increase to 100 °C at a rate of 5 °C/min. The column temperature was subsequently increased to 200 °C at a rate of 20 °C/min. The injector and detector temperatures were set at 220 and 240 °C, respectively. The system was operated in the split injector mode with a split ratio of 9:1. Nitrogen was used as a carrier gas with a flow rate of 25 mL/min, and the injection volume was set at 0.6 µL.

The calibration curve was established based on 12 consecutive injections at six different concentrations (i.e., 0.01, 0.02, 0.05, 0.1, 0.15 and 0.20 mg/mL) of *l*-borneol, and the internal standard methyl salicylate (0.1 mL/mL) is added, respectively. The ratios of peak areas of *l*-borneol and to that of methyl salicylate are used as dependent variable of regression equation, and with the *l*-borneol in different concentrations as independent variable. The *l*-borneol content of samples are expressed as mg of *l*-borneol/g of leaf powder. The relative standard deviations (RSD) were calculated for the relative peak areas of *l*-borneol and methyl salicylate to estimate the precision, repeatability and stability characteristics of this newly developed method. The precision of this new method was also evaluated using intraday variation tests based on six replicate injections of the same sample. The repeatability of this new method was analyzed using six replicate samples. The stability characteristics of the sample solutions were evaluated at different time points during a single 24 h period (0, 2, 4, 8, 12, and 24 h). The recovery efficiency was determined by adding measured amounts of an *l*-borneol standard (5 mg) to an extract of *B. balsamifera* leaves (1 g) with six replicates.

### 3.5. Determination of Total Flavone Content by UV-VIS Spectrophotometry

According to the previous method [16], a small sample (0.5 g) of *B. balsamifera* was extracted under ultrasonic irradiation (40 KHz, 400 W) into a 75% (*v*/*v*) solution of ethanol (25 mL) for 40 min. The resulting extract was filtered through a Millipore filter (0.22 µm, Tianjin Jinteng Laboratory Equipment Co.) before being analyzed by UV-VIS spectrophotometry to determine its total flavone content.

Quantification of total flavone was performed by means of UV-VIS spectrophotometry with chromogenic system of NaNO_2_–Al(NO3)_3_–NaOH [16,18]. Briefly, a small sample (0.5 mL) of filtered extract was transferred to a volumetric flask and the material was diluted to a total volume of 10 mL with 75% (*v*/*v*) ethanol. The solution was then treated with 1 mL of a 5% (*w*/*w*) aqueous NaNO_2_ solution and 1 mL of a 10% (*w*/*w*) aqueous Al(NO_3_)_3_ solution, and the resulting mixture was agitated for 5 min. The mixture was then treated with 10 mL of a 4% (*w*/*w*) aqueous NaOH solution, before being fixed by the addition of 25 mL of 75% (*v*/*v*) ethanol. The mixture was then held for 15 min, before being analyzed on a UV-VIS spectrophotometer (UNICO2012-PCS, Unico Instruments Co., Ltd., Shanghai, China) at 509 nm. Rutin was used as a standard to prepare a calibration curve. The flavone content was then calculated using a linear equation according to the calibration curve.

The calibration curve of the total flavones was established using six different concentrations rutin (0.01, 0.02, 0.04, 0.06, 0.08, and 0.1 mg/mL) as the standard. The absorbance values of total flavones are used as dependent variable of regression equation, with six different concentrations as independent variable. Total flavonoid content is expressed as mg of rutin equivalents/g of leaf powder. The RSD of the absorbance values for the total flavones were calculated to estimate the precision and repeatability of this method, as well as the stability of the samples. The precision of the UV-VIS method was evaluated based on the results of intraday variation tests using six replicate determinations of the same sample. The repeatability of the UV-VIS method was also analyzed using six replicate samples. The stability of the sample solution was evaluated at different time points within an hour period (0, 10, 20, 30, 40 and 50 min). The recovery efficiency was determined by adding a known amount of a rutin standard (30 mg) to an extract of *B. balsamifera* leaves (0.25 g) with six replicates.

## 4. Conclusions

NIR spectroscopy can be used as a rapid, reliable and environmentally friendly method to determine the APIs and the origin of different *B. balsamifera* samples. We have established a diffuse reflectance NIR method for the quantitative determination of the *l*-borneol and total flavone contents of *B. balsamifera*, as well as distinguishing the geographical origins of samples from different regions of China. NIR calibration models of the *l*-borneol and total flavone contents of *B. balsamifera* were established using a PLS method by selecting specific wavelengths using a SiPLS algorithm. These results obtained using these models included the calibration and validation sets and were consistent with the GC and UV-VIS results for the *l*-borneol and total flavone contents of the different *B. balsamifera* samples. According to the model parameters, NIR can be used to detect the *l*-borneol and total flavones contents in *B. balsamifera*. Furthermore, PLS-DA was used to build a model capable of distinguishing the geographical origin of the different samples. The use of a 1D + SG(9) and 2D + SG(9) pretreatment process resulted in a prediction accuracy rate of 100% for the two different geographical origins, demonstrating that PLS-DA may be used to accurately determine the origin of *B. balsamifera*.

## Figures and Tables

**Table 1:** Method parameters and the calibration curves of the reference methods.

Compounds	Ref. Method	Linearity Ranges (μg/mL)	Calibration Curves	R^2^	Precision (RSD%, *n* = 6)	Repeatability (RSD%, *n* = 6)	Stability (RSD%, *n* = 6)	Recovery (%, *n* = 6)
*l*-borneol	GC	10.371–207.428	*Y*_1_ = 14.823*X*_1_ + 0.0129	0.9999	2.10	3.00	0.49	103
Total flavones	UV-VIS	9.176–73.408	*Y*_2_ = 12.847*X*_2_ + 0.0093	1.0000	1.05	3.80	1.89	110

**Table 2:** Statistical results for the *l*-borneol and total flavone contents of *B. balsamifera*.

Compounds	Total Samples			Hainan			Guizhou		
	Content Range (mg/g)	Mean (mg/g)	SD	Content Range (mg/g)	Mean (mg/g)	SD	Content Range (mg/g)	Mean (mg/g)	SD
*l*-borneol	1.00–13.80	5.20	2.60	1.30–12.00	5.30	2.20	1.00–13.80	5.10	3.30
Total flavones	6.60–189.30	61.30	46.20	6.60–189.30	72.20	47.90	8.70–153.30	34.90	29.10

**Table 3:** Performance parameters of the PLS models of the total flavones and *l*-borneol using different spectra pretreatment methods.

Compounds	Pretreatments	Latent Factors	RMSEC	R^2^	Rmsecv	R^2^
Total flavones	Raw	11	0.8258	0.9606	1.1334	0.9278
	SG(9)	11	0.8341	0.9598	1.1349	0.9276
	1D + SG(9)	7	0.8229	0.9609	1.1677	0.9234
	2D + SG(9)	6	0.7269	0.9695	1.6578	0.8457
	MSC	9	1.1210	0.9274	1.4690	0.8788
	SNV	4	1.4127	0.8847	1.5961	0.8569
*l*-borneol	Raw	13	0.1116	0.8056	0.1752	0.5342
	SG(9)	13	0.1179	0.7829	0.1829	0.4919
	1D + SG(9)	14	0.0315	0.9845	0.0917	0.8722
	2D + SG(9)	6	0.0557	0.9515	0.1158	0.7966
	MSC	13	0.1071	0.8210	0.1814	0.5005
	SNV	13	0.1164	0.7886	0.1866	0.4712

Raw: raw spectra; MSC: multiplicative scatter correction; SNV: standard normal variate; SG: Savitzky–Golay filter; 1D: first derivative; 2D: second derivative.

**Table 4:** Performance parameters of the established SiPLS models of the total flavones and *l*-borneol using different spectral pretreatment methods.

Compounds	Pretreatment	Interval Number	Latent Factors	RMSEC	R^2^	RMSECV	R^2^
Total flavones	Raw	7, 12, 16	9	0.9524	0.9476	1.1736	0.9226
	SG(9)	7, 12, 16	9	0.9826	0.9442	1.1836	0.9213
	1D + SG(9)	1, 14, 17	7	1.0445	0.9370	1.3463	0.8982
	2D + SG(9)	3, 7, 15	7	0.5648	0.9816	1.3541	0.8970
	MSC	10, 17, 20	6	1.4587	0.8771	1.7493	0.8281
	SNV	8, 12, 16	5	1.3421	0.8960	1.5071	0.8724
*l*-borneol	Raw	6, 7, 9	13	0.0505	0.9602	0.0822	0.8975
	SG(9)	6, 7, 9	13	0.0612	0.9416	0.0832	0.8948
	1D + SG(9)	6, 7, 10	10	0.0559	0.9511	0.0842	0.8924
	2D + SG(9)	3, 6, 7	6	0.0481	0.9638	0.0812	0.8998
	MSC	6, 7, 9	9	0.0876	0.8803	0.1080	0.8228
	SNV	6, 9, 10	12	0.0442	0.9696	0.0909	0.8744

**Table 5:** PLS-DA classification results obtained using different spectral pretreatment methods.

Pretreatment	Latent Factors	Prediction (%)		
		Total	Guizhou	Hainan
Raw	9	97.30	90.91	100.00
SG(9)	9	97.30	90.91	100.00
1D + SG(9)	8	100.00	100.00	100.00
2D + SG(9)	4	100.00	100.00	100.00
MSC	13	91.89	81.82	96.15
SNV	12	91.89	72.73	100.00

**Table 6:** *B. balsamifera* from different geographical regions.

Sample Codes	Origins	Collection Date (Year/Month)	Sample Codes	Origins	Collection Date (Year/Month)
1–5	Luodian, Guizhou	2013.3	47–52	Danzhou, Hainan	2013.12
6–9	Wuzhishan, Hainan	2013.5	53–55	Luodian, Guizhou	2013.12
10–11	Xingyi, Guizhou	2013.6	56–61	Danzhou, Hainan	2013.4
12	Baise, Guangxi	2013.6	62–67	Danzhou, Hainan	2013.5
13–18	Baisha, Hainan	2013.9	68–73	Danzhou, Hainan	2013.6
19–25	Qiongzhong, Hainan	2013.9	74–79	Danzhou, Hainan	2013.7
26	Xingyi, Guizhou	2013.11	80–85	Danzhou, Hainan	2013.8
27–33	Anlong, Guizhou	2013.11	86–91	Danzhou, Hainan	2013.9
34–36	Ceheng, Guizhou	2013.11	92–97	Danzhou, Hainan	2013.10
37–39	Wangmo, Guizhou	2013.11	98–103	Danzhou, Hainan	2013.11
40–46	Luodian, Guizhou	2013.11	104–109	Danzhou, Hainan	2013.12
